# Long-term reproductive, growth, and carcass performance of Morkaraman, Awassi, and Tuj sheep in semi-arid extensive systems

**DOI:** 10.5194/aab-69-193-2026

**Published:** 2026-03-26

**Authors:** Dogan Turkyilmaz

**Affiliations:** 1 Department of Animal Science, Faculty of Agriculture, Atatürk University, Erzurum 25240, Türkiye

## Abstract

Native sheep breeds are important genetic resources for extensive livestock systems because of their adaptation to extreme conditions and low-input production. However, long-term data on their production performance remains limited. This study aims to provide a comprehensive evaluation of the reproductive traits, growth performance, carcass characteristics, and meat quality of the Morkaraman, Awassi, and Tuj native sheep breeds over a 10-year period under an extensive production system. The research was conducted at the Sheep Husbandry Unit, Food and Livestock Application and Research Center, Atatürk University, Erzurum (39° N, 41° E), Türkiye. The effects of breed, dam age, gender, and type of birth were analyzed using 5000 individual records. Sheep were managed under extensive conditions with flushing diets provided pre-mating, followed by natural mating and pasture-based grazing. Lamb growth performance data were recorded alongside slaughter and carcass traits. Morkaraman and Tuj breeds showed higher litter sizes at birth (1.06 and 1.08, respectively) than Awassi (1.02) ( P < 0.01). Morkaraman and Awassi lambs exhibited superior growth and carcass performance, with birth weights of 4.48 and 4.41 kg, weaning weights of 16.54 and 16.28 kg, slaughter weights of 36.7 and 36.1 kg, hot carcass weights of 16.7 and 16.6 kg, and cold dressing percentages of 47.2 % and 46.6 %, respectively, compared with Tuj lambs (4.05, 15.69, 33.2, 15.2 kg, and 42.7 %). Morkaraman lambs had the highest live weights in all measurement periods ( P < 0.01). In parallel with growth performance, higher carcass trait values were observed in Morkaraman lambs along with Awassi ( P < 0.01). These results suggest that the Morkaraman breed is suitable for weight- and carcass-oriented production for this study. Conversely, the Tuj breed showed comparatively lower carcass fat deposition and fat-related parameters, indicating leaner meat characteristics. This study provides a valuable long-term dataset on the reproductive, growth, and carcass traits that can be useful for improving breed-specific management and breeding programs.

## 1 Introduction

The global consumption of animal products has been increased in parallel with population growth. Over the past decade, the world's red meat supply has increased by over 13 %. This increase in supply is around 10.4 % for bovine meat and 23.3 % for ovine meat. Worldwide, sheep meat production accounts for 5.4 % of the total amount of red meat production, with goat meat accounting for about 3.6 %. In Türkiye, these figures are 24.4 % for sheep meat and 4.8 % for goat meat (FAO, 2024). Türkiye's agricultural industry plays an important role in the national economy, contributing 6.2 % to the total gross national product (TUIK, 2024).

Sheep have multiple economic benefits, serving as a source of income and livestock savings, as well as providing high-quality protein through their milk and meat, along with manure for soil fertilization, which reduces risk and offers valuable wool and skin. Considering the increasing demand for these products due to both population growth and economic concerns, small ruminant production systems need to be managed accordingly. One of the major constraints in production is crossbreeds with adaptation problems, often caused by inadequate livestock breeding management. Native sheep breeds represent valuable genetic resources, characterized by their adaptability to diverse climatic conditions, limited feed availability, and low-input production systems. Sheep breeding methods are initially evaluated using three criteria: reproduction, growth, and survival (Genfors et al., 2023). In this regard, the determination of the current yield characteristics of native breeds that demonstrate superior adaptation to environmental conditions is of critical importance in the delineation of breeding methods, such as crossbreeding, that may be applied to enhance these performances. The enhancement of productivity in breeding systems focused on meat production is dependent upon the selection of breeds with high meat yield or the improvement of reproductive performance by increasing the number of lambs per ewe (Knapik et al., 2017).

In this study, the reproductive traits, growth performance, and meat production characteristics of native sheep breeds were evaluated. The breeds of fat-tailed sheep considered in this study included the Morkaraman, which is a significant population within the indigenous breeds of Türkiye; the Awassi, which is widely raised in Anatolia and the Middle East; and the Tuj sheep, which is an important breed of the Caucasus region (Dagdelen and Esenbuga, 2025).

Although many studies have evaluated reproductive or growth characteristics in native sheep breeds, few have focused multi-characteristic data over a long-term period under extensive conditions. This research is designed to fill this gap by presenting a long-term dataset that includes the entire production cycle – from mating to carcass traits – and provides critical insights into breed-specific performance and sustainable management methods. The objective of this study was to examine the reproductive, growth, and carcass performance of native sheep breeds managed under extensive systems over a decade. By integrating biological and performance data across the full production cycle, this study aims to guide breed-specific selection and sustainable production strategies for semi-arid systems.

## 2 Material and methods

### 2.1 Study area and ethical approval

The research was conducted at the Sheep Husbandry Unit, Food and Livestock Application and Research Center, Atatürk University, Erzurum (39° N, 41° E), Türkiye. All animal procedures were carried out in accordance with the guidelines of the Atatürk University Scientific Research and Publication Ethics Committee on Animal Use (protocol numbers 2016/137 and 2018/64). The sheep used in the study were reared in a location with an average annual precipitation of 431.5 mm, an average temperature of 5.8 °C, and an altitude of 1959 m. The region requires sheep breeds with a high degree of adaptability, given the extremely cold winters ( - 10.2 °C) and the relatively warm summers (19.5 °C). The sheep reared at this unit are kept under conditions similar to those of local flocks in the region, and an extensive breeding system was applied.

### 2.2 Reproductive management and growth recording

Following the pasture period, between October and November, sheep were subjected to flushing feeding for 4 weeks before mating. Sheep were offered 400 g d^−1^ concentrated feeds and 1.5 kg d^−1^ dry hay during the mating and pregnancy periods. The sheep were always provided with water and mineral licks ad libitum. After the flushing process, sheep mated with rams by natural mating for approximately 45 d, during the mating season. The sheep are subdivided into breed groups, with each group comprising an average of 20 ewes and one ram. The rams were selected from the males that were not related to the ewes in the herd and were used for a maximum of 3 years. Subsequently, the lambing season occurred between March and April in different years. Newborn lambs and ewes were moved to a separate pen within 2 h following lambing, and the birth weights were recorded on the same day. Lambs were initially housed in a separate pen with dams for a 3 d period. The lambs with their dams were then transferred to a pen containing a small group of other lambed ewes, where they remained for a period of 2 weeks. Crop feeding was initiated at 50 g d^−1^ per lamb at 1 week of age, and the suckling period was completed when the animals reached an average age of approximately 60 d. The pasture period for lambs began after the weaning weights were recorded. Pasture grazing was conducted in a herd-based manner from the beginning of the June until the end of September. The pasture was characterized by a diverse grassland flora, including species such as *Festuca ovina*, *Agropyron cristatum*, and *Poa pratensis*, which contribute to grazing quality. At the end of the pasture period, the lambs were weighed. In addition, daily live weight gains for the relevant periods were calculated. The study was based on data collected during a 10-year period. During this period (2013–2023), a total of 5000 records were obtained from Morkaraman, Awassi, and Tuj sheep.

### 2.3 Slaughter procedure and carcass measurements

Following the grazing period on pasture, the lambs were fasted for a period for 24 h and weighed, and data recorded as slaughter weights. After removal of the head, skin, feet, and offal at slaughter, the hot carcass weights were recorded. The carcasses were chilled in cold storage at a temperature of + 4 °C for a period of 24 h. After the chilling period, cold carcass weights were determined, and the kidneys, kidney fat, and pelvis fat were removed from the cold carcass and weighed separately. The carcasses were divided into five sections, namely the forearm and flank; leg; rib and loin; neck; and tail. Marbling score, fat thickness, and rib area were measured between the 12th and 13th vertebrae in the *longissimus dorsi* (LD) muscle. Yield grade and proportional yield of boneless retail cuts were calculated using the formula described by Boggs and Merkel (1984).

### 2.4 Meat quality

The pH value and color measurements were taken 24 h postmortem on freshly cut surfaces of the *longissimus dorsi* muscles at uniform anatomical locations. The determination of pH value was conducted using a SCHOTT Lab Star pH meter, and color measurements were taken with a Minolta (CR-400) colorimeter device. All devices were calibrated before analysis according to the manufacturers' recommendations. For CIELAB colorimetry, the values for lightness ( $L^{*}$ ), green-red color ( $a^{*}$ ), blue-yellow color ( $b^{*}$ ), hue angle ( H ), and chroma ( C ) were determined.

For chemical composition analysis, LD muscle samples stored at - 80 °C were analyzed for crude protein, ether extract, and dry matter contents. Crude protein was determined by the Kjeldahl method using approximately 2.5 g of homogenized fresh sample, and nitrogen content was multiplied by 6.25 to obtain crude protein values (AOAC, 2007). Dry matter content was determined using approximately 10 g of sample dried at + 100 °C for 18 h and calculated from the difference between initial and final weights. Ether extract content was determined by the Soxhlet extraction method using dried samples; ether was removed by rotary evaporation, and the remaining fat was weighed (AOAC, 2007). All chemical analyses were performed in duplicate.

Sensory analyses were performed on *longissimus dorsi* muscle samples using a trained panel consisting of 10 experienced panelists. Meat samples stored at - 20 °C were thawed at + 4 °C prior to evaluation. Each sample was cut into slices of approximately 2 cm thickness, placed in heat-resistant cooking bags, and cooked in a water bath until an internal temperature of + 74 °C was reached. Cooked samples were cut into equal-sized portions, coded, and served randomly to the panelists. Between samples, water and unsalted crackers were provided for palate cleansing.

Prior to the experiment, panelists, who had prior experience in meat quality evaluation, participated in orientation and calibration sessions to standardize scoring criteria and ensure consistent evaluation of sensory characteristics. Panelists evaluated flavor intensity (lamb flavor–mutton flavor), tenderness (tender–tough), juiciness (juicy–dry), and overall acceptability (high–low) using a nine-point hedonic scale (1 = lowest, 9 = highest). Warner–Bratzler shear force (WBS) values were determined on cooked samples by cutting perpendicular to the muscle fibers in three replicates, and the results were expressed in pounds. Ensuring the minimization of bias, the samples were coded with random numbers, and the sequence of the samples and the order of service were randomly determined among the panelists and sessions.

### 2.5 Statistics

Statistical analysis of the data was performed using the general linear model (GLM) procedure of the SPSS statistical package (SPSS, 2020). The statistical model for reproductive and growth traits included the fixed effects of breeds (Morkaraman, Awassi, Tuj), dam age (2 to over 7 years), type of birth (single and twin), gender, and year (10 years) according to $Y_{ijklm}=\mu +b_{i}+a_{j}+t_{k}+g_{l}+y_{m}+e_{ijklm}$ , where $Y_{ijklm}$ is the observed value of the dependent variable; μ is the overall mean; $b_{i}$ is the effect of breed; $a_{j}$ is the effect of dam age; $t_{k}$ is the effect of birth type; $g_{l}$ is the effect of lamb gender; $y_{m}$ is the effect of year; and $e_{ijklm}$ is the experimental error.

The statistical model used for slaughter and carcass performance was $Y_{ij}=\mu +b_{i}+s_{j}+y_{m}+e_{ij}$ , in which $s_{j}$ is the effects of slaughtering parameters. Differences among means with P < 0.05 were considered statistically significant. Mean comparisons were performed using Duncan's multiple range test to identify significant differences.

## 3 Results

### 3.1 Reproductive performance

The fecundity and litter size rates at birth, weaning, and the end of the pasture were found to be significantly lower ( P < 0.01) for Awassi ewes compared to Morkaraman and Tuj ewes (Table 1). In consideration of litter size at birth, the ewes aged 5 years demonstrated significantly the highest rates ( P < 0.01), while comparable values were observed in all ewes, apart from the ewes aged 2 years ( P < 0.05), at the weaning and the end of pasture. The results of the study conducted obtained over a 10-year period indicated that the year factor had a significant effect on all the reproductive traits of the ewes. It is possible that differences in climatic conditions, such as temperature and precipitation, as well as herd management factors, including herd size and composition, may have influenced these traits during the study period.

**Table 1:** Effect of breed, age, gender, and type of birth on reproductive performance.

		Fecundity	L-Birth	L-Weaning	L-Pasture
Year		0.003	0.002	< 0.001	< 0.001
Breed		< 0.001	0.001	0.009	< 0.001
	Morkaraman	0.92^a^	1.06^a^	1.01^a^	0.94^a^
	Awassi	0.81^b^	1.02^b^	0.96^b^	0.88^b^
	Tuj	0.91^a^	1.08^a^	1.02^a^	0.93^a^
	SEM	0.016	0.010	0.012	0.014
Age		0.026	0.002	0.008	0.020
	2	0.87^b^	1.02^b^	0.89^b^	0.82^b^
	3	0.89^b^	1.05^b^	1.01^a^	0.93^a^
	4	0.93^a^	1.07^b^	1.03^a^	0.94^a^
	5	0.92^a^	1.12^a^	1.07^a^	0.97^a^
	6	0.94^a^	1.06^b^	1.01^a^	0.93^a^
	7 +	0.90^ab^	1.05^ab^	1.01^a^	0.91^a^
	SEM	0.039	0.047	0.043	0.067

^a,b^ Means with different superscript letters in the same column are significantly different ( P < 0.05).Fecundity: lambing ewes per mated ewes; L-Birth: litter size at birth; L-Weaning: litter size at weaning; L-Pasture: litter size at end of the pasture.

### 3.2 Growth performance of lambs

The statistical analysis showed that the birth weights of lambs born to Morkaraman and Awassi sheep were significantly higher compared to these born to the Tuj breed ( P < 0.01) (Table 2). All breeds exhibited similar live weight gains up to the weaning and end-of-pasture periods; however, Tuj lambs showed lower numerical gains. In addition, the weaning weights obtained from Tuj lambs were lower. Nevertheless, live weight gains were comparable, and Tuj lambs were found to perform as well as Awassi lambs. As expected, the effect of ewe age on lamb growth was significant ( P < 0.05). Lambs born from 4-year-old ewes had the highest weights.

**Table 2:** Effect of breed, dam age, gender, and birth type on growth performance up to end of pasture.

		BW	WW	PW	DWG-W	DWG-P
Year		< 0.001	< 0.001	< 0.001	< 0.001	< 0.001
Breed		< 0.001	< 0.001	0.006	0.178	0.088
	Morkaraman	4.48^a^	16.54^a^	35.84^a^	0.201	0.196
	Awassi	4.41^a^	16.28^a^	33.36^b^	0.198	0.181
	Tuj	4.05^b^	15.69^b^	32.21^b^	0.194	0.176
	SEM	0.029	0.128	0.835	0.006	0.005
Age		0.014	0.013	0.013	0.019	0.097
	2	3.97^b^	14.68^b^	33.25^b^	0.179^b^	0.183
	3	4.36^a^	16.11^ab^	33.09^b^	0.196^ab^	0.180
	4	4.49^a^	17.40^a^	35.82^a^	0.215^a^	0.196
	5	4.27^ab^	16.07^ab^	33.86^ab^	0.197^ab^	0.185
	6	4.39^a^	17.01^a^	34.63^ab^	0.211^a^	0.189
	7 +	4.29^ab^	15.42^ab^	34.16^ab^	0.186^b^	0.187
	SEM	0.119	0.669	1.003	0.008	0.005
Gender		0.052	0.088	0.157	0.309	0.411
	Male	4.45	16.47	34.72	0.201	0.189
	Female	4.16	15.89	33.56	0.196	0.184
	SEM	0.062	0.361	0.502	0.004	0.002
Type of birth		0.001	0.067	0.216	0.305	0.473
	Single	4.37	16.27	34.25	0.198	0.187
	Twin	3.78	15.48	33.21	0.195	0.184
	SEM	0.094	0.491	0.738	0.006	0.004

^a,b^ Means with different superscript letters in the same column are significantly different ( P < 0.05).BW: birth weight; WW: weaning weight; PW: weight at end of the pasture; DWG-W: daily weight gain up to weaning; DWG-P: daily weight gain up to the end of pasture; SEM: standard error of meaning.

### 3.3 Slaughter traits and offal characteristics

Live body measurements and slaughter characteristics indicated that values for Tuj lambs were lower than those for Morkaraman and Awassi ( P < 0.01) (Table 3). Hot and cold carcass weights, and dressing percentages showed similar trends ( P < 0.01). Loss percentages during chilling were significantly higher in Awassi and Tuj lambs compared to Morkaraman lambs ( P < 0.05). Significant differences were observed for some offal parts of lambs. The weight of the four “feet” ( P < 0.01), head, and kidney fat ( P < 0.05) were significantly higher in Morkaraman and Awassi lambs. For other offal parts, such as testicle, spleen, heart, lungs, and liver, no significant differences were found.

**Table 3:** Slaughter traits of Morkaraman, Awassi, and Tuj lambs.

	Breed			SEM	P
	Morkaraman	Awassi	Tuj		
Live body measurements (cm)					
Body length	62.3	60.1	56.8	1.79	0.087
Rump height	64.9	64.7	62.1	0.88	0.293
Shoulder height	63.9	63.6	61.6	1.10	0.316
Chest circumference	76.6	79.4	76.4	1.34	0.639
Chest depth	16.8	17.1	16.6	0.37	0.674
Slaughter and carcass characteristics (kg)					
Slaughter weight	36.7^a^	36.1^a^	33.2^b^	0.61	< 0.001
Hot carcass	16.7^a^	16.6^a^	15.2^b^	0.42	< 0.001
Cold carcass	16.3^a^	16.1^a^	14.7^b^	0.42	< 0.001
Hot dressing (%)	48.5^a^	48.1^a^	43.9^b^	1.22	0.028
Cold dressing (%)	47.2^a^	46.6^a^	42.7^b^	1.21	0.018
Loss (%)	2.63^b^	2.86^a^	2.85^a^	0.047	0.014
Offal parts (kg)					
4 feet	0.768^a^	0.795^a^	0.665^b^	0.018	0.002
Head	2.079^a^	2.015^ab^	1.851^b^	0.065	0.025
Testicles	0.307	0.310	0.254	0.032	0.301
Skin	5.029	5.317	4.764	0.175	0.177
Spleen	0.053	0.054	0.071	0.009	0.664
Heart	0.180	0.189	0.173	0.009	0.557
Lung	0.527	0.497	0.470	0.025	0.228
Liver	0.480	0.452	0.439	0.014	0.154
Kidney	0.083	0.080	0.080	0.002	0.43
Kidney fat	0.115^a^	0.109^a^	0.068^b^	0.011	0.021
Pelvis fat	0.024	0.023	0.012	0.008	0.403

^a,b^ Means with different superscript letters in the same row are significantly different ( P < 0.05); SEM: standard error of meaning; P : significance value.

### 3.4 Carcass characteristics

The carcass components of Morkaraman and Awassi lambs were numerically higher than those of Tuj lambs, except for tail fat (Table 4). However, no statistically significant differences were observed among the breeds except for neck weights ( P < 0.05). For the LD area, fat thickness, yield grade, and retail cuts, the values were slightly higher in Morkaraman and Awassi lambs, but these differences were not statistically significant. Significantly lower values were obtained for the marbling score in Awassi lambs ( P < 0.05). It was observed that Awassi lambs had a short leg width but a longer inner leg length ( P < 0.05). The other measurements of carcasses were comparable among the breeds.

**Table 4:** Carcass characteristics of Morkaraman, Awassi, and Tuj lambs.

	Breed			SEM	P
	Morkaraman	Awassi	Tuj		
Carcass components (kg)					
Forearm and flank	2.79	2.53	2.31	0.107	0.059
Leg	4.64	4.83	4.34	0.186	0.223
Rib and loin	3.91	3.79	3.65	0.139	0.446
Neck	3.01^a^	2.99^a^	2.37^b^	0.143	0.030
Tail fat	1.94	2.01	2.04	0.138	0.856
Carcass quality					
Marbling	2.8^a^	1.3^b^	2.3^ab^	0.281	0.011
LD area (cm^2^)	13.7	13.1	12.5	0.70	0.639
Fat thickness over LD (mm)	2.81	2.37	2.12	0.262	0.274
Yield grade	1.51	1.33	1.23	0.103	0.276
Retail cuts	47.76	47.15	46.9	0.398	0.524
Carcass measurements (cm)					
Carcass length	51.1	49.3	49.7	1.258	0.570
Dorsal-loin length	39.7	39.8	39.8	0.632	0.998
Inner leg length	36.1^b^	37.4^a^	36.0^b^	0.347	0.023
Leg width	20.2^a^	18.3^b^	20.3^a^	0.313	0.013
Leg depth	14.6	14.5	14.0	0.28	0.311
Carcass chest circumference	68.5	69.0	65.6	1.401	0.173
Carcass chest depth	16.8	17.0	16.7	0.437	0.877

^a,b^ Means with different superscript letters in the same row are significantly different ( P < 0.05); SEM: standard error of meaning; P : significance value.

### 3.5 Physical and chemical traits

The pH values of lamb meat did not differ significantly among the breeds ( P > 0.05), with mean values of 5.92, 5.75, and 5.84 for Morkaraman, Awassi, and Tuj lambs, respectively (Table 5). No significant breed effect was observed for color parameters, including lightness ( $L^{*}$ ), redness ( $a^{*}$ ), and yellowness ( $b^{*}$ ) ( P > 0.05). The $L^{*}$ values ranged from 33.16 to 34.27, $a^{*}$ values from 13.84 to 14.71, and $b^{*}$ values from 4.06 to 4.23. Hue angle and chroma values also showed no statistically significant differences among the breeds ( P > 0.05).

**Table 5:** Physical and chemical traits of Morkaraman, Awassi, and Tuj lamb meat.

	Breed			SEM	P
	Morkaraman	Awassi	Tuj		
pH value	5.92	5.75	5.84	0.018	0.287
Color parameters					
$L^{*}$ (lightness)	33.16	34.27	33.71	0.355	0.581
$a^{*}$ (redness)	14.23	13.84	14.71	0.213	0.419
$b^{*}$ (yellowness)	4.23	4.06	4.12	0.192	0.672
Hue angle	16.51	16.78	15.96	0.552	0.358
Chrome value	16.44	16.59	15.61	0.486	0.317
Chemical composition [g (100 g)^−1^]					
Crude protein	22.24	21.66	22.12	0.362	0.106
Ether extract	3.49	3.87	3.15	0.186	0.224
Dry matter	25.78	26.61	25.21	0.419	0.197

SEM: standard error of meaning; P : significance value.

For chemical composition, crude protein content ranged from 21.66 to 22.24 g (100 g)^−1^, ether extract from 3.15 to 3.87 g (100 g)^−1^, and dry matter from 25.21 to 26.61 g (100 g)^−1^. Although Awassi lambs exhibited slightly higher ether extract and dry matter contents, and Morkaraman lambs showed marginally higher crude protein values, these differences were not statistically significant ( P > 0.05). Overall, the physicochemical properties of lamb meat were similar among the three breeds.

### 3.6 Sensory traits

Sensory analyses indicated that meat from Morkaraman, Awassi, and Tuj lambs had similar and moderate scores (Table 6). Sensory trait scores of lamb meat did not differ significantly among the breeds ( P > 0.05). Tenderness scores ranged from 5.3 to 5.8, with the highest value observed in Tuj lambs, followed by Awassi and Morkaraman lambs. Juiciness scores varied between 5.4 and 5.9, with Awassi lambs showing slightly higher values. Flavor scores were very similar among the breeds, ranging from 5.7 to 5.9. Overall acceptability scores were also comparable, varying between 5.6 and 5.8.

**Table 6:** Sensory traits of Morkaraman, Awassi, and Tuj lamb meat.

	Breed			SEM	P
	Morkaraman	Awassi	Tuj		
Tenderness	5.3	5.5	5.8	0.34	0.419
Juiciness	5.5	5.9	5.4	0.28	0.538
Flavor	5.7	5.9	5.9	0.26	0.892
Overall acceptability	5.8	5.6	5.7	0.26	0.869
The number of chews	24.9	25.9	23.1	1.63	0.419
Cooking loss (%)	28.6	29.8	29.3	1.96	0.921
WBS (kg cm^−2^)	4.9	5.2	4.2	0.58	0.426

WBS: Warner–Bratzler shear force; SEM: standard error of meaning; P : significance value.

The number of chews during sensory evaluation ranged from 23.1 to 25.9, with Awassi lambs showing the highest mean value; however, no statistically significant differences were observed among the breeds ( P > 0.05). Cooking loss values varied between 28.6 % and 29.8 %, and these differences were not statistically significant. Warner–Bratzler shear force (WBS) values ranged from 4.2 to 5.2 kg cm^−2^, with Tuj lambs showing slightly lower shear force values; however, the differences among breeds were not significant ( P > 0.05), indicating similar meat tenderness characteristics.

## 4 Discussion

The capacity of reproductive performance to affect production efficiency and the number of lambs born is a significant factor in livestock animal breeding. However, the lifetime performance of the sheep in relation to reproduction is also regarded as a crucial aspect in the lamb production. The study provides valuable insights into the reproductive performances of sheep. Despite the observation of individual variability among ewes, reproductive performance showed no significant differences among breeds, with all breeds in the study exhibiting comparably low levels. These native breeds represent low-input genotypes that have undergone limited selection for reproductive efficiency. The litter size at birth is a crucial factor in evaluating the reproductive performance of sheep. The lambing rates of Morkaraman and Awassi ewes observed in the present study were comparable to those reported by Emsen and Yaprak (2006). In that study, comparable findings for single lambing was also reported, whereas Awassi ewes exhibited a higher rate of twin lambing. The reported results for Morkaraman ewes were similar to the present study. In a study conducted by Kridli et al. (2007), comparable lambing rates were observed in Awassi ewes. The fertility findings at birth and at weaning obtained in this study are consistent with those reported by Abebe et al. (2023) for native breeds. Similarly, Abebe et al. (2023) concluded that fertility traits increase with age, further supporting the findings in this study. The highest survival rates were obtained from Morkaraman and Tuj lambs at weaning and at the end of pasture. Previous studies have indicated that Morkaraman lambs perform the highest performance during the suckling period and the pasture period (Macit et al., 2001, 2002; Kopuzlu and Sezgin, 2017). The study conducted by Türkyilmaz et al. (2017) reported lower fecundity and litter size in Morkaraman sheep, whereas higher litter size was observed in Tuj sheep. These findings also indicate that variations between years, as well as differences in climatic conditions and herd management, played a significantly role in affecting reproductive performance.

**Figure 1:**
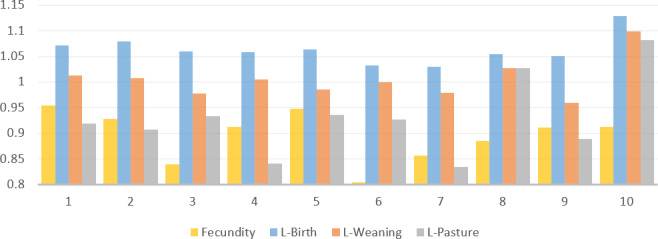
Time series of annual means for reproductive traits. Fecundity: lambing ewes per mated ewes; L-Birth: litter size at birth; L-Weaning: litter size at weaning; L-Pasture: litter size at end of the pasture.

Indeed, the reproductive performance of sheep may be influenced by a number of factors, including breed, climatic conditions, health, nutrition, and systems of management. Following a 10-year observational study, it can be stated with certainty that the native breed of sheep used in the study did not display the characteristics associated with prolificacy (Fig. 1). Considering that fat-tailed breeds like Morkaraman, Awassi, and Tuj, reared in similar locations, have presented relatively higher reproductive traits in previous studies (Abdel-Rafa et al., 2022; Aygün and Çelikyürek, 2020; Trabzon and Öztürk, 2019), these breeds may show potential for improvement in reproductive traits with suitable management and breeding strategies.

**Figure 2:**
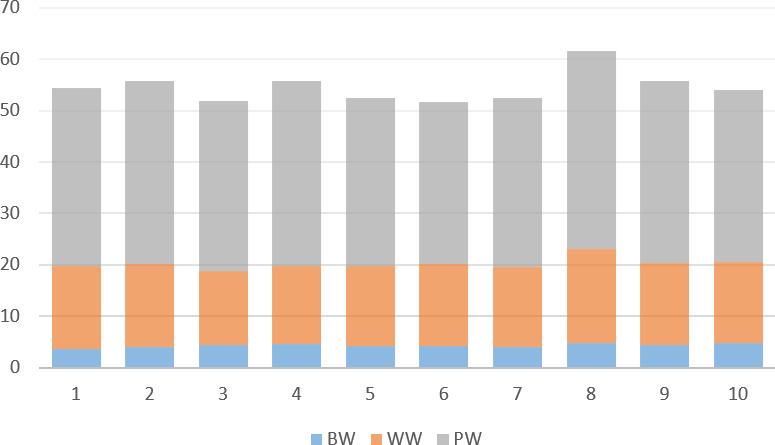
Time series of annual means for growth traits. BW: birth weight; WW: weaning weight; PW: weight at end of the pasture.

The growth performance of native lambs is dependent upon their genetic adaptation to the local environmental conditions and traditional management practices. The analysis of growth parameters, such as birth weight, weaning weight, and average daily gain, offers insights into the developmental norms, adaptability, and suitability of the lambs for extensive production systems. It is therefore crucial to understand the growth performance of these lambs in order to develop effective selection and breeding strategies, such as improving weight gain, feed efficiency, and overall productivity. The availability of such information is fundamental to the development of sustainable breeding practices and the improvement of lamb productivity, particularly in systems where native breeds achieve optimal growth under low-input, traditional management conditions. The study shows that breed, dam age, gender, and type of birth significantly affect the lamb growth, with the Morkaraman breed performing marginally higher in weight gain. The ewes aged 4–6 years generally gave birth to lambs with higher weights. It is possible that the ewes aged 2 years were unable to provide adequate milk for their lambs during their first lambing season compared to older ewes. As expected, the effect of ewe age on growth performance of the lambs was determined to be statistically significant ( P < 0.05). In particular, the lambs born from ewes aged 4 years showed the highest mean live weights. In comparison with the growth performance of native breed lambs stated in varies studies (Bilgin et al., 2004; Bozgüllü and Macit, 2022; Dagdelen and Esenbuga, 2024; Khan et al., 2020; Meddah et al., 2024), the current study observed that all lamb breeds performed at a similar or higher result. Furthermore, the observed birth weight was found to be lower than the reported for Norduz lambs of the native breed by Karakuş and Atmaca (2016). Wanjala et al. (2023) reported that native breeds showed higher growth performance than a meat-type breed as an indicator of early tolerance and adaptation to local environmental variables. In the same study, it was proposed that native breeds may benefit genetically from in-breed selection. The results indicated a significant effect of the year on growth performance. It is clear that the results of this 10-year study were significant in determining the characteristics of native breeds, considering the different growth performance results obtained in different years (Fig. 2). As a consequence, the results obtained in the majority of studies conducted with domestic breeds have reported notable differences. The growth performance of native breeds is closely correlated with their adaptation capability to local environmental conditions. The findings of this study indicate that the Morkaraman and Awassi breeds have developed traits that enable them to persist under traditional management practices. This adaptability is crucial for sustainable sheep production.

The success of lamb meat production is directly correlated with the carcass weight and dressing percentage. The slaughter weights observed in the study are consistent with those reported by Macit et al. (2001) for Morkaraman, Awassi and Tuj lambs under similar conditions. Dressing percentage is one of the principal parameters used for evaluating carcass characteristics, and it has considerable economic importance. Dressing percentages in the study are consistent with that reported by Esenbuga et al. (2009). It has been reported in various studies that dressing percentages of lambs fed on pasture were lower than those of lambs fed concentrate (Karaca et al., 2016; Priolo et al., 2001; Turkyilmaz et al., 2024). The observed values in the study indicated that Morkaraman and Awassi lambs, which are commonly raised under extensive management systems, showed similar meat production efficiencies among the fat-tailed breeds. In this regard, it was observed that the dressing percentages of the lambs fed pasture in this study were lower than the values reported for lambs fed concentrate. The differences in dressing percentages can be explained by the breed effect but also by carcass weight and offal parts of the carcass. This difference in the observed findings could be attributed to the gastrointestinal contents and the energy level of feeding. The similar performance of Morkaraman and Awassi breeds in terms of slaughter and carcass characteristics suggest their suitability for domestic and export markets where larger carcass weights are preferred. The Tuj breed could be a suitable alternative for consumers of low-fat meat products because of their leaner profiles. In addition, cooling loss percentage is dependent on the thickness of the fat covering the carcasses (Suliman et al., 2021). It can also be explained by observation that Tuj sheep have slightly lower values for fat thickness. The offal parts of the carcass showed lower weights compared to that reported for the native Norduz breed in similar regions (Karaca et al., 2016). The findings in this study underline the importance of aligning breed selection with specific market demands, as also emphasized in studies on carcass characteristics by Esenbuga et al. (2009).

It is commonly stated that the stress experienced during slaughter has a significant effect on the pH value of meat. Differences in post-mortem muscle pH are generally attributed to variation in the pre-slaughter stress levels (Stewart et al., 2018). However, the comparable pH values observed in this study indicate that the stress factor was consistent across all lamb breeds.

The results of several studies conducted with fat-tailed breeds indicate that pH values were within the expected range, whereas the results for color values were comparatively low (Karaca et al., 2016; Macit et al., 2001). The pH values for all lamb meats were found to be comparable to that reported by Atti and Mahouachi (2011). However, color values – particularly lightness, redness and yellowness – were slightly lower than the values reported in that study. The pH value is a significant factor affecting meat color. A higher pH value causes a darker meat color, which means lower lightness parameters (Priolo et al., 2001). Comparable to previously studies (Karaca and Kor, 2015; Karaca et al., 2016), the lambs in this study had high a pH value and lower lightness. It was expected that the redness ( $a^{*}$ ) was similar in all lambs, given that the $a^{*}$ value is related to the pigmentation in the meat, which is affected by the live weights of the lambs (Ripoll et al., 2008). In a study conducted by Hopkins et al. (2007) on the acceptability criteria for lamb meat color, it was determined that an $L^{*}$ value of 34–35 and an $a^{*}$ value of 9.5 represented the threshold for acceptability. The results obtained in this study indicated that the $L^{*}$ value was comparable to and slightly below the stated threshold. Furthermore, the $a^{*}$ value, which indicated it may be more suitable for evaluating consumer color acceptability, was found to be above the threshold value.

The chemical composition of meat is a significant determinant of quality. The dry matter, protein, and ether extract values of meat were found to be higher than the values reported by Karaca et al. (2016) for native Norduz breed and Kawecka and Radkowska (2018) for the native Swiniarka breed. Sañudo et al. (2016) reported a higher ether extract in meat for Rasa Aragonesa than those obtained in this study. The fat content of meat has been demonstrated to play a significant role in influencing consumer preferences. A study has indicated that a fat content in meat of between 3 % and 6 % has a positive effect on consumer preference (Miller, 2002). These characteristics are of critical importance for consumer satisfaction. The values obtained for all breeds in this study align with studies indicating that intramuscular fat contributes to flavor perception (Wood et al., 2004; Zhang et al., 2022).

The quality of lamb meat is influenced not only by physical and chemical traits but also by sensory attributes. The sensory characteristics of lamb meat play a significant role in determining consumer acceptance. It is essential to consider a range of factors, including tenderness, juiciness, flavor, and overall acceptability in order to better understand the sensory traits of lamb meat, as well as cooking loss, WBS, and number of chews. The results of this study are consistent with those of previous studies on Australian lambs (Hopkins et al., 2007), in that the sensory traits of the Morkaraman, Awassi, and Tuj breeds demonstrated consistent consumer preference patterns. The Tuj lambs showed a marginally higher tenderness score in alignment with the WBS force value, thereby indicating a softer meat. The Tuj sheep breed displayed a relatively superior tenderness, which could be an advantage in specific market sectors that demand softer meat textures. The Awassi lambs exhibited slightly higher scores for juiciness and flavor, which may indicate a potentially superior intramuscular fat content. However, the observed differences were not statistically significant. The slightly higher juiciness and flavor scores of Awassi lambs may appeal to consumers who prefer a higher level of taste intensity.

## 5 Conclusion

The present study provides long-term performances, including the entire production cycle of the Morkaraman and Awassi sheep breeds, which constitute a majority of the sheep population in Türkiye, as well as the Tuj sheep, characterized by similar morphological and physiological traits, which are comparatively less numerous. The findings indicate that Morkaraman sheep exhibited higher growth and carcass yield performance, whereas Tuj sheep, despite their smaller population size, demonstrated comparable reproductive efficiency and leaner carcass profiles. These results highlight the significance of breed-specific data in informing sustainable breeding and management strategies. Also, these findings support targeted breeding and management strategies that align with market demands and ecological limitations in semi-arid regions. The study provides baseline phenotypic insights to support informed management and the optimization of decisions relating to native sheep breeding, particularly in extensive systems facing environmental constraints and the growing demand for efficient meat production.

## Data Availability

The original data used in this study can be obtained from the corresponding author upon reasonable request with official permission.
